# A new blood parasite of leaf warblers: molecular characterization, phylogenetic relationships, description and identification of vectors

**DOI:** 10.1186/s13071-018-3109-9

**Published:** 2018-10-04

**Authors:** Carolina Romeiro Fernandes Chagas, Dovilė Bukauskaitė, Mikas Ilgūnas, Tatjana Iezhova, Gediminas Valkiūnas

**Affiliations:** Institute of Ecology, Nature Research Centre, Akademijos 2, 21, LT-09412 Vilnius, Lithuania

**Keywords:** *Haemoproteus*, New species, *Haemoproteus homopalloris* n. sp., Phylogenetic relationships, *Culicoides*, Vectors, Sporogony

## Abstract

**Background:**

Blood parasites of the genus *Haemoproteus* Kruse, 1890 are cosmopolitan, might be responsible for mortality in non-adapted birds, and often kill blood-sucking insects. However, this group remains insufficiently investigated in the wild. This is particularly true for the parasites of leaf warblers of the Phylloscopidae Alström, Ericson, Olsson & Sundberg the common small Old World passerine birds whose haemoproteid parasite diversity and vectors remain poorly studied. This study reports a new species of *Haemoproteus* parasitizing leaf warblers, its susceptible vector and peculiar phylogenetic relationships with other haemoproteids.

**Methods:**

Wood warblers (*Phylloscopus sibilatrix* Bechstein) were caught in Lithuania during spring migration, and blood films were examined microscopically. Laboratory reared *Culicoides nubeculosus* Meigen were exposed experimentally by allowing them to take blood meals on one individual harbouring mature gametocytes of the new *Haemoproteus* species (lineage hPHSIB2). To follow sporogonic development, the engorged insects were dissected at intervals. The parasite lineage was distinguished using sequence data, and morphological analysis of blood and sporogonic stages was carried out. Bayesian phylogeny was constructed in order to determine the phylogenetic relationships of the new parasite with other haemoproteids.

**Results:**

*Haemoproteus* (*Parahaemoproteus*) *homopalloris* n. sp. was common in wood warblers sampled after arrival to Europe from their wintering grounds in Africa. The new parasite belongs to a group of avian haemoproteid species with macrogametocytes possessing pale staining cytoplasm. All species of this group clustered together in the phylogenetic analysis, indicating that intensity of the cytoplasm staining is a valuable phylogenetic character. Laboratory-reared biting midges *C. nubeculosus* readily supported sporogony of new infections. Phylogenetic analysis corroborated vector experiments, placing the new parasite in the clade of *Haemoproteus* (*Parahaemoproteus*) parasites transmitted by biting midges.

**Conclusions:**

*Haemoproteus homopalloris* n. sp. is the third haemoproteid, which is described from and is prevalent in wood warblers. Phylogenetic analysis identified a clade containing seven haemoproteids, which are characterised by pale staining of the macrogametocyte cytoplasm and with ookinetes maturing exceptionally rapidly (between 1 to 1.5 h after exposure to air). Both these features may represent valuable phylogenetic characters. Studies targeting mechanisms of sporogonic development of haemoproteids remain uncommon and should be encouraged. *Culicoides nubeculosus* is an excellent experimental vector of the new parasite species.

## Background

Blood parasites of the genus *Haemoproteus* Kruse, 1890 (Haemosporida: Haemoproteidae) are distributed worldwide and are among the most extensively studied blood parasites of birds, particularly in the temperate regions [1]. They are transmitted by biting midges (Ceratopogonidae) and louse flies (Hippoboscidae) [2]. *Haemoproteus* species were considered relatively benign to their avian hosts [3]. However, several recent studies demonstrated the negative influence of these parasites and even mortality due to haemoproteosis not only in non-adaptative birds [4–11], but also in blood-sucking insects [12, 13].

Numerous studies addressed morphological and molecular characterization, distribution and genetic diversity of haemoproteids [14–20]. However, some bird groups remain insufficiently investigated. This is particularly true for leaf warblers of genus *Phylloscopus* Boie belonging to the Phylloscopidae Alström, Ericson, Olsson & Sundberg. The wood warbler, *Phylloscopus sibilatrix* Bechstein, is a common European passerine bird wintering in sub-Saharan Africa [21]. Despite its broad range of occurrence, only two haemoproteid species, *Haemoproteus majoris* Laveran, 1902 [2, 22–24] and *Haemoproteus belopolskyi* Valkiūnas, 1989 [25], have been reported in this bird.

Several research groups successfully used molecular techniques to detect haemoproteids in wild caught insects [26–31]. PCR-based protocols detect the presence of parasite DNA in the insect, but do not provide information on which development stage the parasite is present. In other words, these tools are insufficiently sensitive to conclude if invasive stages (sporozoites) develop in the PCR-positive insect and if insects can act as vectors and transmit the parasites [32]. Observation of infective sporozoites in salivary glands strongly suggests the vectorial capacity of blood-sucking insects. Experimental infections provide the opportunity to follow parasite development and to morphologically characterize each life stage [33, 34].

Studies addressing the sporogonic development and transmission of avian *Haemoproteus* species are few [29, 35–38]. Vector species and complete life-cycle remains unknown for the great majority of *Haemoproteus* parasites and their lineages [1, 38, 39]. *Culicoides* species have been used in experimental studies addressing parasite development [33, 34]. These insects are abundant in mixed forest zone in eastern Europe [37]. Despite of their diminutive size and difficulties to maintain in colonies [40], *Culicoides nubeculosus* Meigen and several other species have been kept in laboratory colonies successfully [41, 42]. *Culicoides nubeculosus* has been used to study the sporogonic development of *Haemoproteus handai* Maqsood, 1943 [43], *Haemoproteus tartakovskyi* Valkiūnas, 1986 [34], *Haemoproteus noctuae* Celli & Sanfelice, 1891 and *Haemoproteus syrnii* Mayer, 1910 [33].

During this study, we discovered a new *Haemoproteus* species that infects wood warblers. This parasite is described using morphology of blood stages and molecular data of partial cytochrome *b* (*cytb*) gene sequence. To access information about sporogonic development, we experimentally infected the biting midge *Culicoides nubeculosus.* The main objectives of this study were (i) to characterize the new *Haemoproteus* species morphologically; (ii) to develop its molecular characterization based on partial *cytb* sequence; (iii) to determine phylogenetically closely related parasite species; and (iv) to follow sporogonic development in experimentally infected vector.

## Methods

### Collection and examination of bird blood samples

We collected blood samples from 16 adult wood warblers (*Phylloscopus sibilatrix*) at the Ornithological Station in Ventės Ragas, Lithuania (55°20'28.1"N, 21°11'25.3"E) during May in 2015, 2016 and 2017. The birds were caught with mist nets. Approximately 30 μl of blood was withdrawn from the brachial vein using a sterile syringe needle and capillary tubes. Several drops were used immediately for preparation of blood smears on three glass slides, and the remaining blood was stored in SET buffer (0.05 M tris, 0.15 M NaCl, 0.5 M EDTA, pH 8.0) for molecular diagnostics. Blood smears were air-dried, fixed with absolute methanol, and stained with Giemsa [44]. Preparations of good quality and sufficient parasite intensity with single infection, as determined both by microscopic examination and PCR-based testing, were used for morphological characterization of the new species.

Olympus BX41 microscope equipped with PixeLINK and imaging software Megapixel FireWire Camera Release 3.2 were used to examine the blood films and prepare illustrations. Measurements were taken from the images using the calibrated Motic Images Plus 2.0. The slides were examined for 15–20 min at low magnification (×400), and then at least 100 fields were studied at high magnification (×1000). Parasite identification follows the guidelines of Valkiūnas [2]. All measurements are given in micrometres. Images of positive preparations were collected for measurement. Representative preparations were deposited in the Nature Research Centre, Vilnius, Lithuania (accession number 49021 NS and 49022 NS). The analyses were carried out using the Statistica 7 package. A parahapantotype blood film with gametocytes of closely related haemoproteid *Haemoproteus palloris* Dimitrov, Iezhova, Zehtindjiev, Bobeva, Ilieva, Kirilova, Bedev, Sjohölm & Valkiūnas, 2016 (deposited at Nature Research Centre, Vilnius, accession number 48832 NS) was used for comparisons with the new *Haemoproteus* species.

Parasitemia was estimated as a percentage by actual counting the number of parasites per 1000 red blood cells or per 10,000 red blood cells during light infections (i.e. < 0.1%) [45]. Morphology of gametocytes of the new species was also compared with the type-specimens deposited in Institute of Nature Research Centre, Vilnius, Lithuania, of *Haemoproteus majoris* (accession number 48893 NS, from *Phylloscopus trochilus* Linnaeus) and *Haemoproteus belopolskyi* (accession number 435.85p, from *Hippolais icterina* Vieillot) the only two *Haemoproteus* parasites reported in *P. sibillatrix* so far. Hapantotype material of the new species was also compared with other *Haemoproteus* species with pale staining macrogametocytes: *Haemoproteus pallidus* Valkiūnas & Iezhova, 1991 (accession number 963.89, from *Ficedula hypoleuca* Pallas), *Haemoproteus pallidulus* Križanauskienė, Pérez-Tris, Palinaukas, Hellgren, Bensch & Valkiūnas, 2010 (accession number 5420 NS, from *Sylvia atricapilla* Linnaeus), *Haemoproteus minutus* Valkiūnas & Iezhova, 1992 (accession number 245.85p, from *Turdus merula* Linnaeus), *Haemoproteus concavocentralis* Dimitrov, Zehtindjiev, Bensch, Ilieva, Iezhova & Valkiūnas, 2014 (accession number 48756 NS, from *Coccothraustes coccothraustes* Linnaeus) and *Haemoproteus vacuolatus* Valkiūnas, Iezhova, Loiseau, Chasar, Smith & Sehgal, 2008 (accession number 42415 NS, from *Andropadus latirostris* Strickland).

### Sporogonic development experimental design

A naturally infected wood warbler with single infection with the new *Haemoproteus* lineage was used as a donor of gametocytes to expose *Culicoides nubeculosus* biting midges. The presence of a single infection in the donor bird was confirmed by microscopic examination (see above) and PCR-based testing, as described below. Insects were reared in the laboratory according to Boorman et al. [41]. Experimental procedures were according to Bukauskaitė et al. [13]. Briefly, biting midges were kept in card boxes covered with fine mesh bolting silk. For the experiment, a box with unfed insects was gently pressed to the feather-free area on pectoral muscles of infected bird. *Culicoides nubeculosus* took blood meals through the bolting silk, with the great majority of females being fully engorged within 30–40 min. Then, biting midges were transferred to a bigger cage made of bolting silk (12 × 12 × 12 cm), males and non-fed females were removed. Remaining insects were kept in a room with controlled temperature (22° C), relative humidity (75 ± 5%) and light-dark photoperiod of 17:7 h. Insects were supplied with a 10% sugar solution offered in cotton pads.

### Dissection of biting midges, preparations of parasites and microscopic examination

Experimentally exposed biting midges were dissected, and preparation of ookinetes, oocysts and sporozoites were made. First, the insects were anesthetized by placing them in a tube covered with cotton-wool pads moistened with 96% ethanol. Biting midges were dissected on intervals in order to follow the development of the parasite in the insect. We examined midgut contents for ookinetes 0.5–12 h post exposure, midgut wall for oocysts 2–5 days post exposure (dpe), and salivary glands for sporozoites 7–8 dpe.

For visualizing ookinetes, midgut was dissected and gently crushed on the slide; the preparations were fixed and stained the same way as blood films. For oocyst observation, temporary preparations were made. Midguts were isolated on a glass slide and a drop of 2% mercurochrome solution was placed on the guts, which was then covered with coverslips. This simplified observation of oocysts. To visualize sporozoites, preparation was made by extracting the salivary glands from biting midges and gently crashing them to prepare small thin smears, which were fixed with absolute methanol and stained with 4% Giemsa solution for 1 h. After each insect dissection, residual parts of their bodies were fixed in 96% ethanol and used for PCR-based analysis to confirm presence of corresponding parasite lineage in vectors. Dissected needles were disinfected in fire to prevent contamination after each dissection.

All vector preparations were examined using Olympus BX43 light microscope equipped with Olympus SZX2-FOF digital camera and imaging software QCapture Pro 6.0, Image Pro Plus (Tokyo, Japan). All preparations were examined as described above for blood smears, and the representative preparations were deposited in the Nature Research Centre, Vilnius, Lithuania (accession numbers 49023 NS and 49024 NS).

### DNA extraction, PCR, sequencing and phylogenetic analysis

Blood samples from the donor bird and residual parts of infected biting midges were examined for haemosporidian parasites by PCR amplification. Total DNA was extracted from both materials using a standard ammonium acetate method [46] and quantified by NanoPhotometer® P330 (IMPLEN). A nested PCR protocol was used to amplify a *cytb* gene fragment [47, 48]. The first pair of primers (HaemFNI/HaemNR3) amplifies sequences of *Plasmodium*, *Haemoproteus* and *Leucocytozoon*. The second pair of primers (HaemF/HaemR2) is specific for *Plasmodium* and *Haemoproteus* parasites. We performed PCR amplification in 25 μl total volume including 50 ng of total genomic DNA template (2 μl), 12.5 μl of Phusion High-Fidelity PCR Master Mix (Thermo Fisher Scientific, Vilnius, Lithuania), 8.5 μl nuclease-free water and 1 μl of each primer (10 μM concentration). One positive (infection confirmed by microscopy analysis) and one negative control (ultrapure water) were used. Positive results were visualized by electrophoresing 2 μl of the final PCR product on a 2% agarose gel. Amplicons of proper length (approximately 500 bp) were precipitated and sequenced from both ends using Big Dye Terminator V3.1 cycle Sequencing Kit and ABI PRISM^TM^ 3100 capillary sequencing robot (Applied Biosystems, Foster City, California). Sequences were edited and aligned using the BioEdit program [49] and deposited in the GenBank database (accession number MH513601). The presence of double peaks in sequence chromatograms was considered a co-infection.

A Bayesian phylogeny of parasite lineages was constructed based on alignment of 45 *cytb* lineages (33 *Haemoproteus* spp. and 11 *Plasmodium* spp.) using MrBayes version 3.2 [50]. One lineage of *Leucocytozoon* sp. (lineage lSISKIN2) was used as the outgroup. All lineages were carefully selected based on studies that provided morphological identification of parasites. We used the General Time Reversible model (GTR) selected by the software jModelTest 2 [51] as the best-fit model under the Bayesian Information Criterion. Gaps and missing data in the alignment were discarded prior to analyses. Two simultaneous runs were conducted with a sample frequency of every 100th generation over 3 million generations. We discarded 25% of the trees as ‘burn in’ period. The remaining trees were used to construct a majority rule consensus tree. The phylogenies were visualized using Fig Tree 1.4 [52]. The codes of c*ytb* lineages are given according to MalAvi database, with a letter ‘h’ starting codes of *Haemoproteus* spp. lineages and a letter ‘p’ starting codes of *Plasmodium* spp. lineages. The sequence divergence between different lineages was calculated using Jukes-Cantor model of substitution, with all substitutions weighted equally (uniform rates), implemented in the program MEGA7 [53].

## Results

### Microscopic and molecular analysis of blood samples

In all, 43.8% of wood warbler tested by PCR and microscopy were infected with *Haemoproteus* parasites. All infections were detected by both methods equally. We reported two different lineages of *Haemoproteus* species in tested birds: hPHSIB1 (*Haemoproteus majoris*) and hPHSIB2 (*Haemoproteus homopalloris* n. sp.). The prevalence of infection with lineage hPHSIB2 was 31%. Two individuals harboured double haemosporidian infection, one with two *Haemoproteus* species, and another one with *Haemoproteus* and *Plasmodium* species. These double infections were detected also by PCR.

**Fig. 1:**
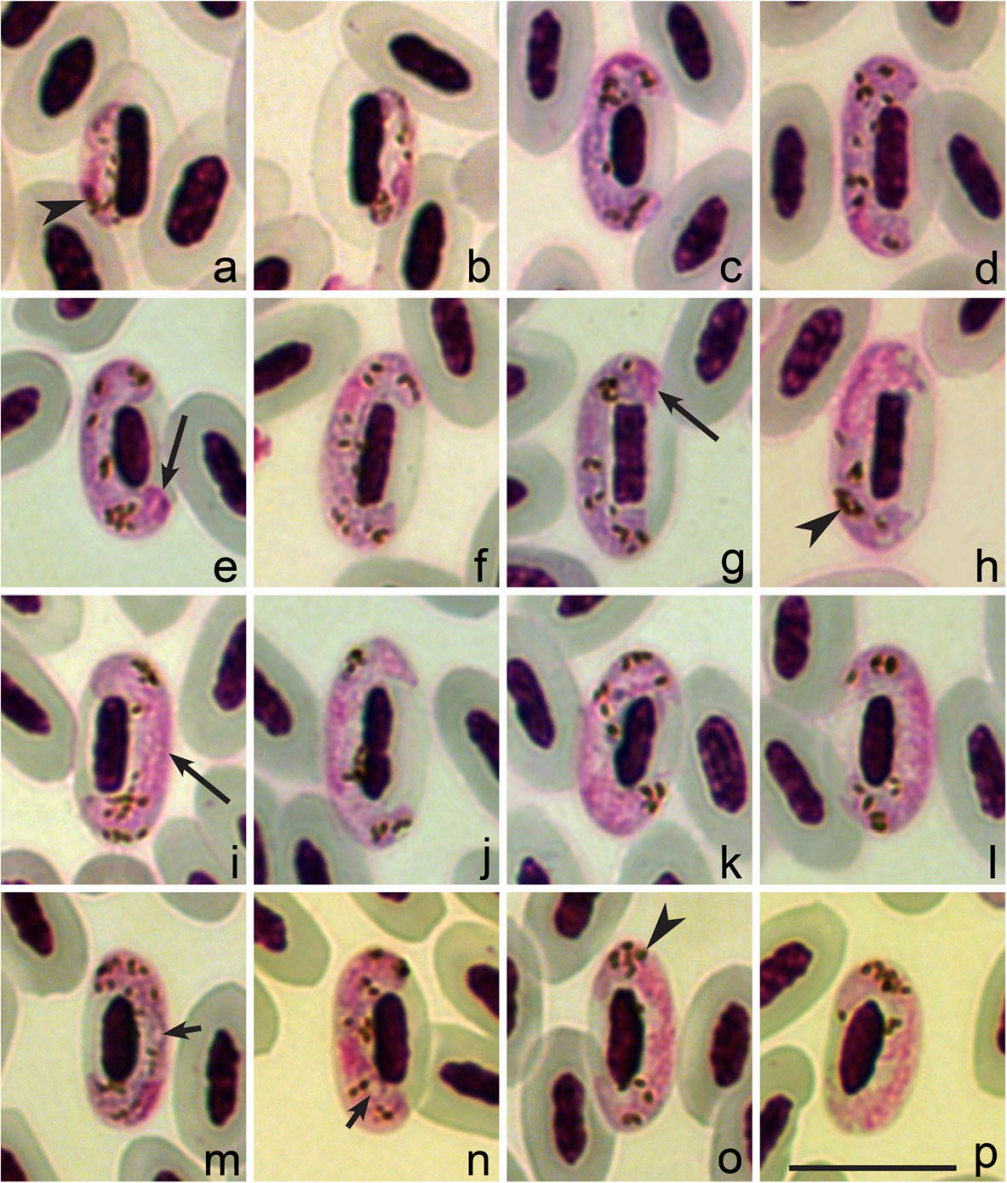
Gametocytes of two species of haemoproteids described from leaf warbles, Phylloscopidae. *Haemoproteus homopalloris* n. sp. (**a**-**l**) and *Haemoproteus palloris* (**m**-**p**). Young gametocytes (**a**, **b**), macrogametocytes (**c**-**g**, **m**, **n**) and microgametocytes (**h**-**l**, **o**, **p**). Long arrows: gametocyte nuclei; short arrows: vacuole-like spaces in macrogametocytes; arrowheads: pigment granules. Giemsa-stained thin blood films. *Scale-bar*: **a**-**p**, 10 μm

**Fig. 2:**
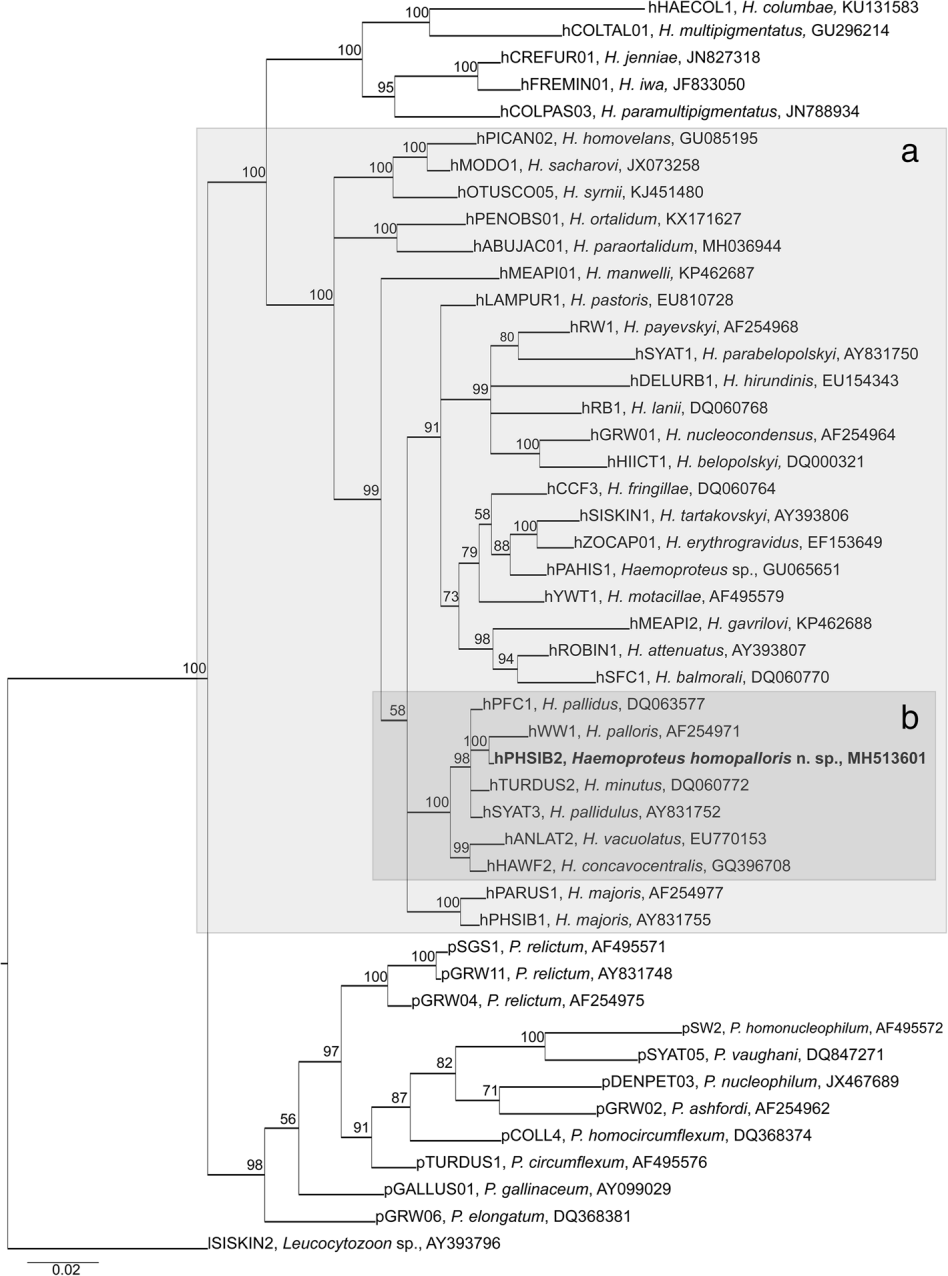
Bayesian phylogenetic inference of *cytb* gene lineages (479 bp) of 35 *Haemoproteus* spp. The tree is rooted with *Leucocytozoon* sp. (lineage lSISKIN2). Clades A and B indicate species of the subgenus *Parahaemoproteus* (**a**) and haemoproteids with pale-staining cytoplasm of gametocytes (**b**). MalAvi lineage codes are provided, followed by parasite species names and GenBank accession numbers. Nodal support values indicate Bayesian posterior probabilities. New species is given in bold

**Fig. 3:**
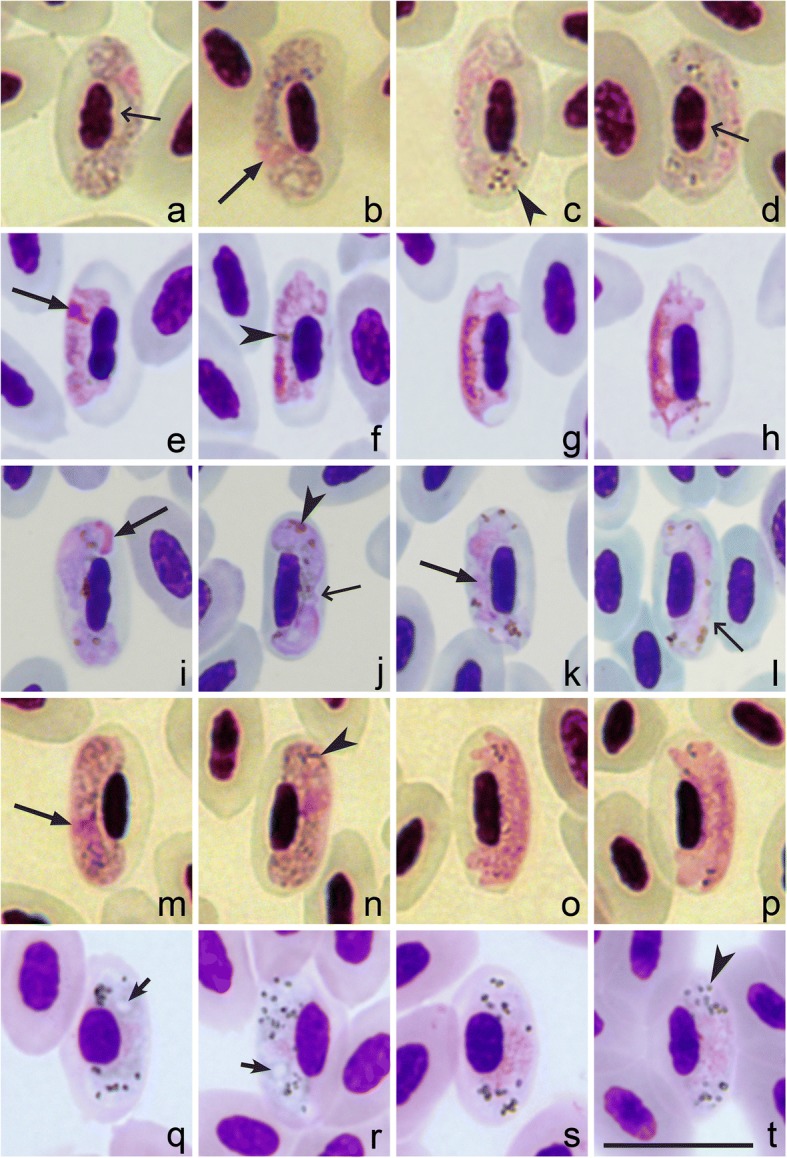
*Haemoproteus* spp. with pale staining of macrogametocyte cytoplasm. *Haemoproteus concavocentralis* (**a**-**d**), *H. minutus* (**e**-**h**), *H. pallidus* (**i**-**l**), *H. pallidulus* (**m**-**p**) and *H. vacuolatus* (**q**-**t**). Macrogametocytes (**a**, **b**, **e**, **f**, **i**, **j**, **m**, **n**, **q**, **r**), microgametocytes (**c**, **d**, **g**, **h**, **k**, **l**, **o**, **p**, **s**, **t**). Note the following valuable diagnostic features of the parasites: presence of a space between the nucleus of the infected erythrocyte and the growing gametocyte in *H. concavocentralis* (**a**); clearly irregular outline of mature gametocytes, which do not touch the poles of infected erythrocytes in *H. minutus* (**e**-**h**); gametocyte which are closely appressed to the nucleus of erythrocyte but do not touch the envelope of erythrocyte along their entire margin in *H. pallidus* (**j**, **l**); small pigment granules in mature gametocytes of *H. pallidulus* (**m**-**p**); presence of one prominent vacuole in the cytoplasm of each advanced macrogametocyte in *H. vacuolatus* (**q**-**t**). All these features are not characteristics of *H. homopalloris* n. sp. (see Fig. 1). Long simple arrows: gametocyte nuclei; short simple arrows: vacuole-like spaces in macrogametocytes; arrowheads: pigment granules; long simple wide arrows: space present between the parasite and an infected erythrocyte nucleus (**a**, **d**) and space between the parasite and the envelope of infected erythrocyte (**j**, **l**). Giemsa-stained thin blood films. *Scale-bar*: **a**-**t**, 10 μm

Phylogenetic analysis confirmed that morphological characteristics of gametocytes are of phylogenetic value because the lineage hPHSIB2 was readily distinguishable both morphologically (Fig. 1) and in the tree (Fig. 2). The lineage hPHSIB2 clustered with other so-called pale staining haemoproteid species, with a high (100%) posterior probability (Fig. 2, clade B): *Haemoproteus pallidus*, *H. palloris*, *H. minutus*, *H. pallidulus*, *H. vacuolatus* and *Haemoproteus concavocentralis*. It is worth noting that the mean genetic distance among *cytb* lineages within this clade was low (1.5%), but all parasites in this clade have unique morphological characters, based on which they can be distinguished from each other (see Fig. 3 and the Remarks below).

ᅟ

**Family Haemoproteidae Doflein, 1916**

**Genus** ***Haemoproteus*** **Kruse, 1890**

ᅟ

***Haemoproteus*** **(** ***Parahaemoproteus*** **)** ***homopalloris*** **n. sp.**

ᅟ

***Type-host*:** *Phylloscopus sibilatrix* Bechstein, 1793 (Passeriformes, Phylloscopidae), wood warbler.

***Type-locality*:** Ornithological Station in Ventės Ragas (55°20'28.1"N, 21°11'25.3"E), Lithuania.

***Type-specimens*:** Hapantotypes (accession numbers 49021 NS and 49022 NS, adult bird *Phylloscopus sibilatrix*; parasitaemia 0.1%, 5.vi.2017, Ornithological Station Ventės Ragas, collected by M. Ilgūnas) were deposited in the Institute of Ecology of Nature Research Centre, Vilnius, Lithuania. Parahapantotype (accession number G466204, other data as for the hapantotype) was deposited in the Queensland Museum, Brisbane, Australia. Co-infection with microfilaria is present in the type-material.

***Site of infection*:** Mature erythrocytes; no other data.

***Prevalence*:** 31% (5 out of 16 examined wood warblers were infected).

***Representative DNA sequence*:** Mitochondrial *cytb* lineage hPHSIB2 (478 bp, GenBank accession number MH513601).

***Vector*:** Sporogony completed and sporozoites developed in experimentally infected biting midges *Culicoides nubeculosus.* This insect is a convenient experimental vector. Natural vectors remain unknown. Representative preparations of sporogonic stages are deposited in the Institute of Ecology of Nature Research Centre, Vilnius, Lithuania, with the accession numbers of 49023 NS and 49024 NS.

***ZooBank registration*:** To comply with the regulations set out in article 8.5 of the amended 2012 version of the *International Code of Zoological Nomenclature* (ICZN) [54], details of the new species have been submitted to ZooBank. The Life Science Identifier (LSID) of the article is urn:lsid:zoobank.org:pub:AC9794B3-D735-4D36-BB6E-5CDF1CF2BA3F. The LSID for the new name *Haemoproteus* (*Parahaemoproteus*) *homopalloris* is urn:lsid:zoobank.org:act:5481116F-3F96-40D2-956E-96C710C64F29.

***Etymology*:** The species name refers to the morphological and morphometric similarity of the new species with *Haemoproteus palloris*, a closely related haemoproteid infecting a closely related avian host, the willow warbler *Phylloscopus trochilus*.

### Description (Fig. 1a-l, Table 1)

***Young gametocytes*.** Rarely seen in the type-material (Fig. 1a, b). Elongate, with even outline and prominent pigment granules. Develop in mature erythrocytes; advanced growing gametocytes closely adhere to erythrocyte nuclei and extend longitudinally along nuclei.

**Table 1:** Morphometric data (in μm) for host cells, mature gametocytes and sporozoites of *Haemoproteus homopalloris* n. sp., lineage hPHSIB2

Feature	Range (Mean ± SD) (*n* = 21)
Uninfected erythrocyte	
Length	10.9–12.8 (11.6 ± 0.6)
Width	5.6–6.5 (6.1 ± 0.3)
Area	48.7–64.3 (57.2 ± 4.2)
Uninfected erythrocyte nucleus	
Length	5.0–6.6 (5.8 ± 0.4)
Width	1.9–2.5 (2.2 ± 0.1)
Area	9.4–14.4 (11.6 ± 1.5)
Macrogametocyte	
Infected erythrocyte	
Length	11.0–13.0 (12.1 ± 0.4)
Width	5.2–6.6 (6.0 ± 0.3)
Area	53.1–65.6 (60.3 ± 3.8)
Infected erythrocyte nucleus	
Length	5.0–7.2 (6.2 ± 0.5)
Width	1.7–2.5 (2.1 ± 0.2)
Area	9.6–16.6 (11.5 ± 1.5)
Gametocyte	
Length	13.2–17.9 (15.1 ± 1.2)
Width	1.8–2.8 (2.4 ± 0.3)
Area	30.7–39.4 (35.9 ± 2.7)
Gametocyte nucleus	
Length	1.8–3.6 (2.5 ± 0.5)
Width	0.9–2.8 (1.4 ± 0.5)
Area	1.9–5.1 (3.3 ± 0.8)
Pigment granules	12–20 (15 ± 1.8)
NDR	0.6–1.1 (0.8 ± 0.1)
Microgametocytes	
Infected erythrocyte	
Length	11.3–14.0 (12.4 ± 0.6)
Width	5.2–6.6 (6.1 ± 0.4)
Area	53.9-74.3 (62.7 ± 5.0)
Infected erythrocyte nucleus	
Length	5.3–7.2 (6.1 ± 0.5)
Width	1.7–2.5 (2.1 ± 0.2)
Area	10.4–13.8 (11.4 ± 0.9)
Gametocyte	
Length	14.8–18.0 (16.4 ± 0.9)
Width	1.9–2.9 (2.3 ± 0.3)
Area	31.0–43.1 (38.3 ± 2.3)
Gametocyte nucleus ^a^	
Length	–
Width	–
Area	–
Pigment granules	9–18 (13 ± 2.6)
NDR	0.6–1.0 (0.8 ± 0.1)
Sporozoites	
Length	6.4–10.0 (7.9 ± 1.1)
Width	1.1–1.9 (1.5 ± 0.2)
Area	6.9–12.0 (9.1 ± 1.5)

^a^Gametocyte nucleus was not well defined and is difficult to measure

*Abbreviations*: *NDR* nucleus displacement ratio according to Bennett & Campbell (1972), *SD* standard deviation

ᅟ

***Macrogametocytes*.** Develop in mature erythrocytes. Cytoplasm staining pale-blue, heterogeneous in appearance, lacking volutin granules. Outline even or slightly wavy (Fig. 1c-g). Vacuoles or vacuole-like spaces absent in the cytoplasm. Gametocytes grow along nuclei of infected erythrocytes, enclose nuclei with their ends, but do not encircle them completely (Fig. 1c-g). Advanced and fully grown macrogametocytes closely appressed both to nuclei and envelop of host cell. Fully grown gametocytes fill erythrocytes up to their poles, not displacing or only slightly displacing nuclei of infected cells laterally. Parasite nucleus relatively small (Table 1), of variable form and position; usually in subterminal position in gametocytes (Fig. 1c, d, f), but also observed in strictly terminal position (Fig. 1e, g) in 12% of macrogametocytes, a characteristic feature of this species development. Nucleolus not seen. Pigment granules roundish or oval, predominantly of medium size (0.5–1.0 μm), usually randomly scattered throughout the cytoplasm. Influence of gametocytes on host cell is non-pronounced (Table 1).

ᅟ

***Microgametocytes*.** General configuration as in macrogametocytes with the usual haemosporidian sexual dimorphic characters, i.e. with large diffuse nuclei and relatively pale staining of the cytoplasm (Fig. 1h-l). Outline often even, but markedly irregular terminal gametocyte edges were also commonly observed (Fig. 1h).

### Remarks

So far, *H. homopalloris* has only been recorded in *Phylloscopus sibilatrix*. One sequence with 100% similarity is deposited in GenBank (accession KJ488925), it was also reported in *P. sibilatrix*, in Western Greater Caucasus [55].

A characteristic feature of *H. homopalloris* n. sp. is the relatively pale staining of the cytoplasm in macrogametocytes, so that macro- and microgametocytes are relatively poorly distinguishable based on this character (compare Fig. 1f and Fig. 1i or l). Gametocytes with pale staining cytoplasm have been described in several species of *Haemoproteus* [2, 56–58]. These parasites seem to be common in African birds and can be often encountered in migrant European birds wintering in Africa [2, 56–58]. The pale staining cytoplasm is particularly different in cases of co-infections with two species (one with pale-stained and second with dark-stained macrogametocytes) present in same blood films.

Among the haemoproteids of passerine birds, *H. homopalloris* n. sp. is most similar to *Haemoproteus palloris*, lineage hWW1 (Fig. 1m-p). These parasites develop in closely related avian hosts, the wood warbler and the willow warbler *Phylloscopus trochilus*, so should be distinguished. They can be differentiated due to the presence of vacuoles or vacuole-like spaces in the majority (80%) of the advanced macrogametocytes of *H. palloris* (Fig. 1m, n) whereas such structures are absent in gametocytes of *H. homopalloris* n. sp. The second taxonomically distinctive character is the pattern of growth of gametocytes in these two parasites. In *H. palloris* (Fig. 1o), an unfilled space is usually present between the gametocyte and the erythrocyte nucleus [15]. This is not characteristic for *H. homopalloris* n. sp. The presence of nuclei in strictly terminal position in 12% of *H. homopalloris* magrogametocytes is another difference between these two species since this has not been reported in *H. palloris*. Despite of the presence of distinguishing morphological features, gametocytes of *H. palloris* and *H. homopalloris* n. sp. are similar (compare Fig. 1c-l with Fig. 1m-p) and the genetic difference among these two lineages is small (1.1% or 5 bp in 479 bp of the *cytb* gene sequence).

**Fig. 4:**
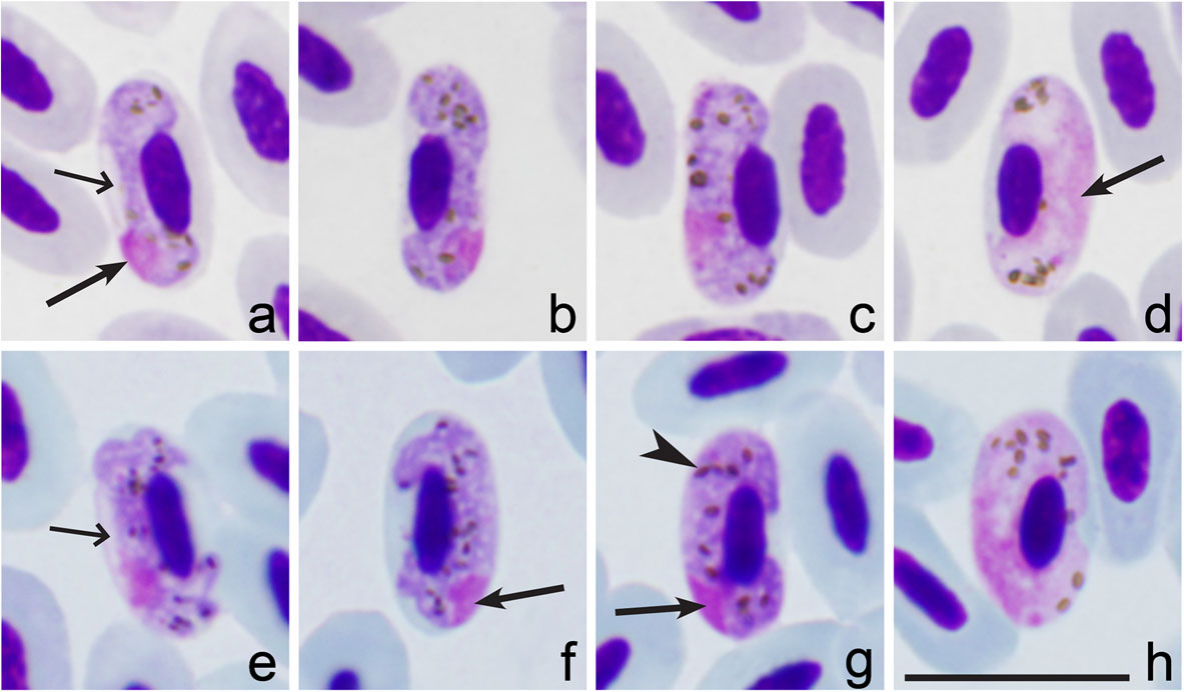
Gametocytes of two species of haemoproteids, which have been reported in the wood warbler *Phylloscopus sibilatrix*. Macrogametocytes (**a**-**c**, **e**-**g**) and microgametocytes (**d**, **h**) of *Haemoproteus majoris* (**a**-**d**) and *H. belopolskyi* (**e**-**h**). Note that the intensity of staining of the cytoplasm is different in macro- and microgametocytes. Long arrows: gametocyte nuclei; short arrows: vacuole-like spaces in macrogametocytes; arrowheads: pigment granules. Giemsa-stained thin blood films. *Scale-bar*: **a**-**h**, 10 μm

*Haemoproteus majoris* (lineage hPHSIB1) has been often reported in wood warblers [22–24]. Phylogenetic analysis showed that *cytb* lineage hPHSIB1 is significantly divergent from the lineage hPHSIB2 of *H. homopalloris* n. sp. (4.5% difference in 17 bp of *cytb* gene fragment) and parasites are morphologically different (compare Fig. 1c-l with Fig. 4a-d). The most distinctive difference between *H. majoris* and *H. homopalloris* is the presence of dumbbell-shaped growing gametocytes in the former ([2], see Fig. 4a), but such gametocytes are absent in *H. homopalloris* n. sp. Additionally, fully grown gametocytes of *H. majoris* markedly displace host cell nuclei laterally (NDR = 0.4 ± 0.1) [2], but this is not a case for *H. homopalloris* n. sp. (NDR = 0.8 ± 0.1) (compare Fig. 1c-l with Fig. 4b-d).

*Haemoproteus homopalloris* n. sp*.* (hPHSIB2) can be also readily distinguished from other *Haemoproteus* species with pale staining macrogametocytes. *Haemoproteus concavocentralis* (hHAWF2) has a characteristic space between nucleus of infected erythrocyte and parasite in growing gametocytes [58] (see Fig. 3a-d). *Haemoproteus minutus* (hTURDUS2) possesses a clearly irregular outline, and it does not touch the poles of infected erythrocytes [2, Fig. 3e-h]. *Haemoproteus pallidus* (hPFC1) gametocytes are closely appressed to the nucleus of erythrocyte but usually do not touch the envelope of erythrocyte along their entire margin [2, Fig. 3i-l]. Gametocytes of *Haemoproteus pallidulus* (hSYAT3) possess small pigment granules even in mature gametocytes ([57], Fig. 3m-p). *Haemoproteus vacuolatus* (hANLAT2) possesses one prominent vacuole in the cytoplasm of each advanced macrogametocyte ([56], Fig. 3q, r). None of these features are characters of *H. homopalloris* n. sp*.*

### Sporogony in biting midges

**Fig. 5:**
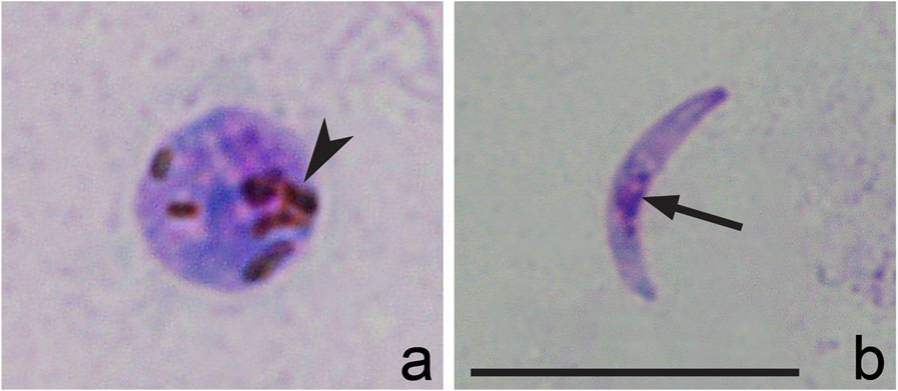
Sporogonic stages of *Haemoproteus homopalloris* n. sp. in the biting midge *Culicoides nubeculosus*. Zygote (**a**) and sporozoite (**b**). Arrowhead: pigment granules; arrow: sporozoite nucleus. Methanol-fixed and Giemsa-stained thin films. *Scale-bar*: **a**, **b**, 10 μm

The presence of numerous sporozoites in salivary glands confirms that *H. homopalloris* n. sp*.* can complete sporogony in *C. nubeculosus*. Ookinetes were not reported in preparations, but zygotes were numerous 8 h post-exposure (Fig. 5a). Oocysts were seen in midgut in temporary preparations 4 dpe. Sporozoites were detected in salivary glands preparations 7 dpe (Fig. 5b). The sporozoites possess fusiform bodies with centrally located nuclei and approximately equally pointed ends (Fig. 5b, Table 1). The PCR-based analysis and sequencing confirmed the presence of hPHSIB2 lineage in experimentally infected biting midges at sporozoite stage.

## Discussion

*Haemoproteus homopalloris* n. sp. is the third species of haemoproteid parasite identified in wood warblers. The infection prevalence was high in the studied bird population. It is interesting to note that all birds were caught with stationary mist nets. In other words, the birds flew in the nets themselves, so they were actively moving individuals and looked healthy from this point of view.

The new species clustered with seven *Haemoproteus* species, which all possess the pale staining cytoplasm in macrogametocytes in the phylogenetic analysis based on *cytb* data (compare Figs. 1 and 3 with Fig. 4). This is not characteristic of the great majority of haemosporidian parasites, in which the cytoplasm of macrogametocytes stains more intensively than in microgametocytes (Fig. 4) [59].

The pale staining of the cytoplasm in *H. homopalloris* n. sp. (Fig. 1) and related species (Fig. 3) might be due to the approximately similar density of organelles in the cytoplasm of macrogametocytes in comparison to microgametocytes, resulting in accumulation of similar amount of stain in both types of cells during staining procedures. Additional electron microscope studies are needed to test this hypothesis. Different cellular structure of macro- and microgametocytes can be explained from the point of view of life span of these cells. Numerous cell organelles are needed for a long survival of macrogametocyte cells, which are relatively long-lived cells in comparison to microgametocytes. After fertilization, each macrogametocyte, transforms to zygote and then the same cell develops into ookinete and further, to relatively long living oocyst [2]. Microgametocytes are relatively short-lived cells; they only produce microgametocytes, which develop within several minutes in vectors. This suggests that haemoproteids with pale staining of the macrogametocyte cytoplasm should develop fast during sporogony in comparison to the species with intense staining of the cytoplasm. Available experimental observations support this and show that complete ookinete formation of *H. minutus* and *H. pallidus*, which macrogametocytes are pale-stained (Fig. 1c-l and Fig. 3), occur within 1 to 1.5 h both *in vitro* and *in vivo* at 18 to 20° C [2, 38]. These parasites also have tiny ookinetes (approximately 10 μm in length), which probably facilitates their rapid movement. The biological meaning of this phenomenon remains unclear. Because pale staining parasites cluster together in the phylogenetic tree (Fig. 2), it is possible that the rapid development of ookinetes might also occur in all other species in this clade. There is no explanation why this pattern is so applicable for the group of pale staining haemoproteid parasites. However, this pattern does not apply for other species like *H. majoris*, *Haemoproteus tartakovskyi* and *H. belopolskyi*, in which macrogametocytes are densely stained with Giemsa (Fig. 4) and mature ookinetes develop relatively slowly (between 6–24 h at the same conditions) [2, 38].

Morphological descriptions of *Haemoproteus homopalloris* n. sp. species was accompanied by DNA sequence information and phylogenetic hypothesis for its relationship with already described parasites. It has been suggested [60] and then confirmed by several other studies [15, 58, 61] that *Haemoproteus* lineages, which differ over 5% in partial *cytb* sequence (479 bp), are likely to be morphologically differentiated and can be distinguished on gametocyte stage. However, it was also shown that some *cytb* lineages of *Haemoproteus* spp. with difference of just two nucleotides (or 0.7%) could be identified based on morphological features of their gametocytes ([56, 62]; this study). This is particularly recognisable in closely related species (Figs. 1 and 3) with pale staining of macrogametocyte cytoplasm [15, 56–58]. The greatest divergence among the seven described species of this group is 3.1%.

The lineage hPHSIB2 of *H. homopalloris* n. sp. clustered with other lineages of the subgenus *Parahaemoproteus* (Fig. 2, clade A) and with other pale staining parasites (Fig. 2, clade B). Interestingly, this group of parasites forms a congruent monophyletic group with low *cytb* genetic divergence (up to 3.1%), but with clearly distinguishable morphological features (Fig. 3). The majority of haemoproteid species with pale staining of macrogametocytes have been reported in European birds wintering in sub-Saharan Africa or resident bird species in this region [2, 15, 56–58]. It might be that they originally evolved in countries with warm climates where they are diverse. Interestingly, *H. homopalloris* n. sp. and *H. minutus* readily complete sporogony in European biting midges at relatively low temperatures (close to 20° C) ([38]; this study), an indication that there are no obstacles to their transmission in the Palaearctic from the point of view of vector availability and temperature needed for sporogony.

It is important to note that one species of haemoproteid with pale staining cytoplasm in macrogametocytes, *H. minutus*, causes mortality of captive parrots in Europe [9, 63]. The mortality is due to heart damage by tissue exo-erythrocytic meronts of this parasite, which abort development before development of parasitemia [11]. *Haemoproteus minutus* is broadly distributed in the Palaearctic region, and several closely related lineages of this pathogen have been identified [63]. It is possible that other closely related to *H. minutus* parasites of clade B (Fig. 2) also might be virulent in non-adapted avian hosts. It is not clear if *H. homopalloris* n. sp. is virulent as the former species; the pathology of this parasite should be investigated in the future.

In this study, two birds were co-infected with different haemosporidian species, and the double infections were detected by both PCR diagnostics and microscopic examination. Various combinations of mixed infections are common in wild naturally infected birds [58, 64–68]. It is important to note that presence of mixed infections often markedly vary among different bird populations. Such infections might predominate and represent more than 80% in some bird species [64]. Mixed infections have been reported to be highly virulent in avian hosts [68, 69]. However, the currently used general primers often underestimate the prevalence of mixed infections [65, 70, 71], and this calls for application of new PCR protocols, which are sensitive to detect mixed infections of *Haemoproteu*s and *Plasmodium* parasites [72]. However, all currently available PCR-based protocols remain insensitive to read mixed infections of several haemosporidian species belonging to the same genus or subgenus, suggesting essential need of application of microscopic examination and morphological identification in parasite biodiversity studies in the wild [44].

Four *Haemoproteus* species can complete sporogony in *C. nubeculosus*. These are *Haemoproteus handai, Haemoproteus noctuae, Haemoproteus syrnii* and *Haemoproteus tartakovskyi* [33, 34, 43]. This study adds *H. homopalloris* n. sp. to this list of haemoproteid species transmitted by this biting midge and reinforces the importance of *C. nubeculosus* in experimental vector research. The pattern of sporogony in *Culicoides impuctatus* seems to be similar in different *Haemoproteus* species under experimental conditions. During sporogonic development of *H. balmorali* Peirce, 1984, *H. belopolskyi*, *H. dolniki* Valkiūnas & Iezhova, 1992, *H. fringillae* Labbé, 1894, *H. lanii* Mello, 1936, *H. minutus*, *H. parabelopolskyi* Valkiūnas, Križanauskienė, Iezhova, Hellgren & Bensch, 2007 and *H. tartakovskyi* ookinetes can be reported between 6 h and 3 dpe and sporozoites between 5 and 7 dpe, indicating asynchronous development ([2, 33, 38]; this study). In *C. nubeculosus* sporogony of *H. homopalloris* n. sp., *H. noctuae*, *H. syrnii* and *H. tartakovskyi* occurs at similar rates: oocysts can be seen 3 dpe until 5 dpe, and sporozoites about 7 dpe ([33, 34]; this study). Interestingly, the sporogony rate of *Haemoproteus* species inhabiting phylogenetically distantly related birds belong to different orders (Passeriformes and Strigiformes) is similar in same species of biting midges ([33, 34]; this study).

The presence of few oocysts in temporary preparations was likely due to the low parasitemia in the donor bird during this study (0.1%). Heavy *Haemoproteus* parasitemia is markedly virulent in blood-sucking insects, and such infections might kill them due to the damage by numerous migrating ookinetes [12]. When performing experimental infections with vectors it is important to use donor birds with light *Haemoproteus* sp. parasitemia (about 0.1%). If parasitemia is high (≥ 1%) in the blood meal, mortality is usually high in biting midges and other blood-sucking insects, resulting in rapid death of experimental groups [13]. Thus, donor birds should be carefully selected for experimental vector research. Interestingly, mainly light parasitemia (< 1%) usually is present in wild-caught birds [16, 19, 58, 73, 74], and such infections are particularly important for transmission. This explains the biological meaning of persistence of haemosporidian parasites in birds at stage of low parasitemia from the viewpoint of parasite transmission.

## Conclusions

*Haemoproteus homopalloris* n. sp. (lineage hPHSIB2) is the third haemoproteid species reported in wood warblers. The new species belongs to the subgenus *Parahaemoproteus* based on the morphology of the sporogonic stages, susceptibility of *Culicoides* biting midges and phylogeny. The phylogenetic analysis identified a well-supported clade containing seven haemoproteids, including the new species, which possess pale staining macrogametocytes and develop exceptionally rapidly at ookinete stage; biological meaning of these characters remains unclear. Studies targeting mechanisms of sporogonic development of haemoproteids and other wildlife haemosporidian parasites remain uncommon and should be encouraged. *Culicoides nubeculosus* is an excellent experimental vector of *H. homopalloris* n. sp. and several other avian haemoproteids. This insect is relatively easy to rear, and it is recommended in laboratory experimental research.
